# Supplementary material for: Chromatin architecture sets origin licensing capacity
Source: bioRxiv. 2026 May 28:2026.05.25.727720. Preprint. [Version 1] doi: 10.64898/2026.05.25.727720 (PMC13232315; doi:10.64898/2026.05.25.727720)
Supplement: Supplement 2 [file NIHPP2026.05.25.727720v1-supplement-2.pdf]

Supplemental Figures

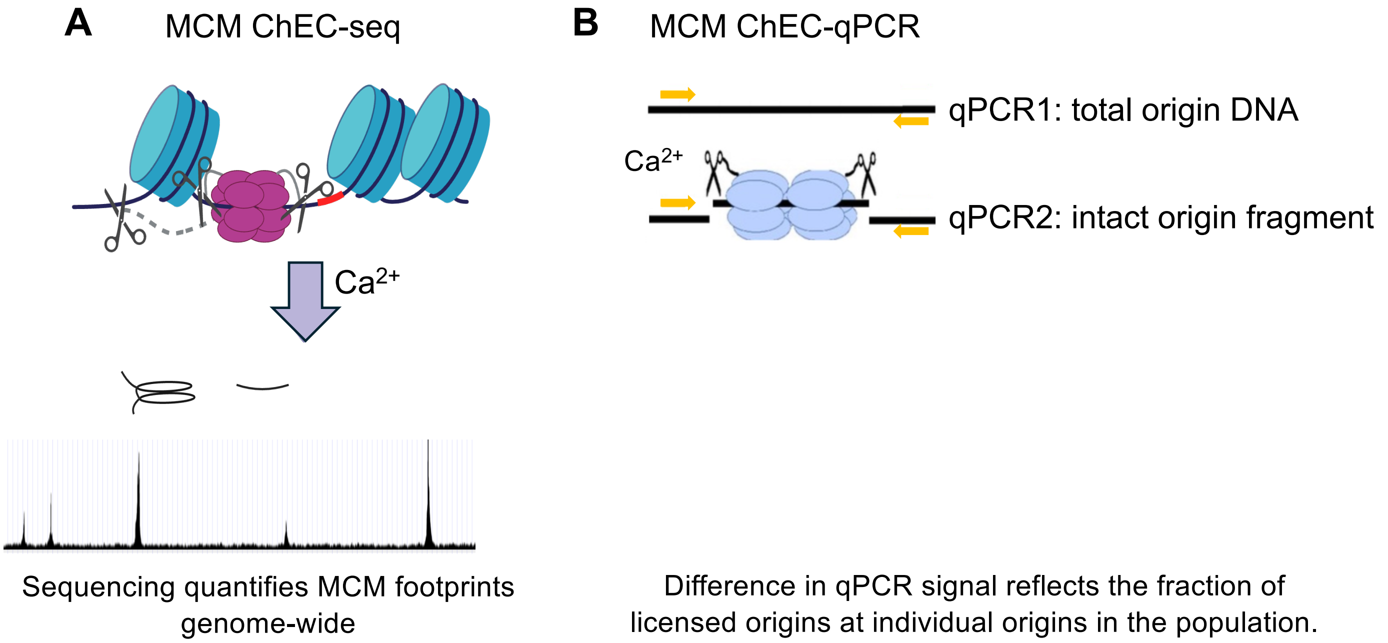

Figure S1. Principle of the MCM–ChEC assays used to measure origin licensing.

(A) MCM–ChEC-seq. Micrococcal nuclease (MNase) fused to an MCM subunit cleaves DNA adjacent to loaded MCM double hexamers upon addition of Ca^{2+}. Sequencing of the resulting fragments quantifies MCM footprints genome-wide, providing a measure of helicase loading at replication origins. Libraries were prepared without size selection, ensuring retention of small MNase fragments; larger fragments are disfavored during library preparation due to their size.

(B) MCM–ChEC-qPCR. MNase cleavage at licensed origins disrupts the PCR amplicon spanning the cleavage site. Quantitative PCR using origin-specific primers amplifies only intact origin DNA; cleavage therefore reduces the qPCR signal in proportion to the fraction of origins that have undergone MNase cleavage. Because MNase cleavage occurs only at origins that have loaded an MCM double hexamer, the decrease in qPCR signal provides a quantitative measure of the fraction of licensed origins in the population.

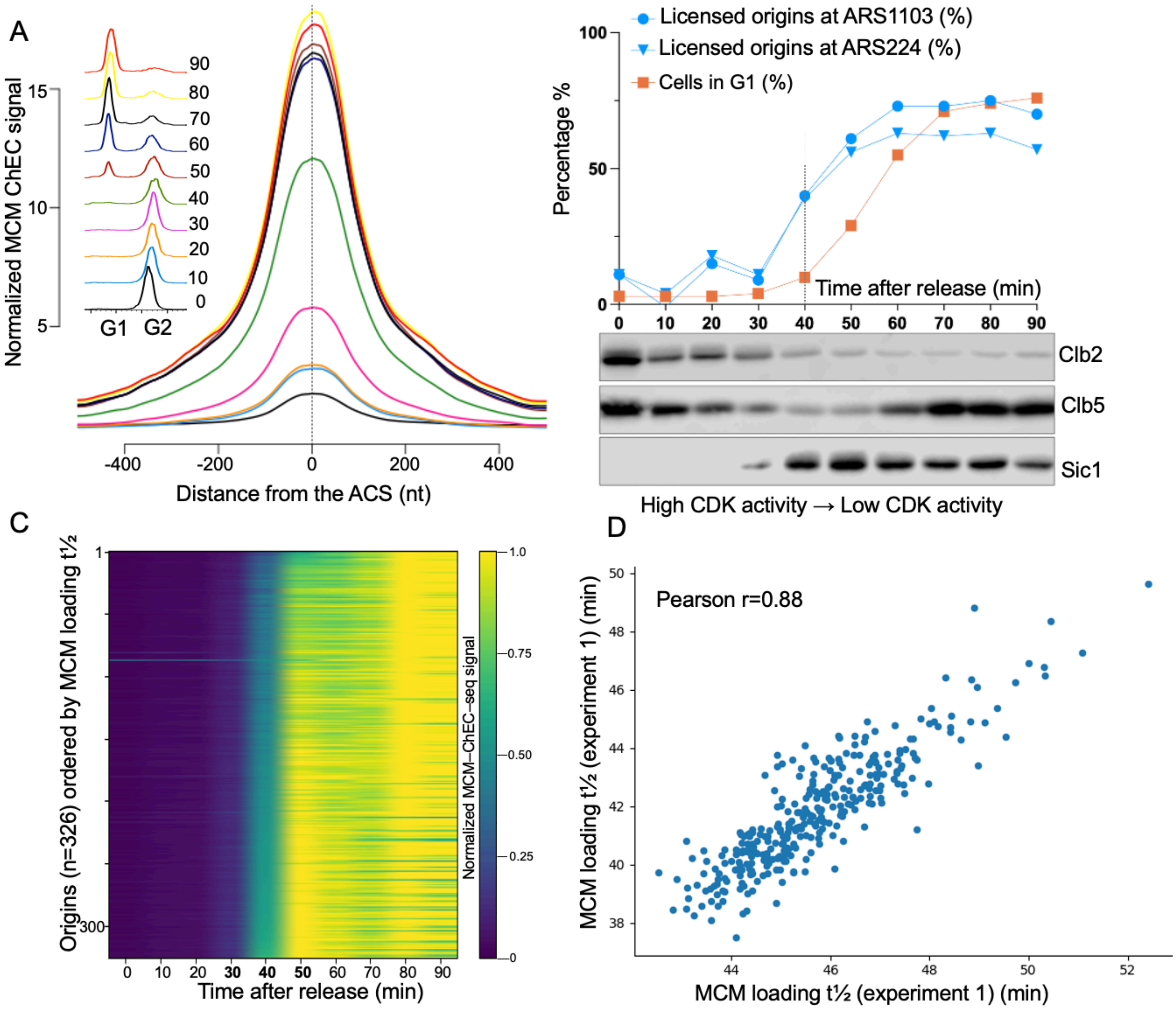

Figure S2. Independent replicate of the replication origin licensing time course.

(A) Aggregate MCM–ChEC–seq signal across origins following release from nocodazole arrest. Normalized MCM–ChEC signal averaged across 326 annotated replication origins and aligned relative to the ACS is shown for the indicated times after release. As observed in the experiment shown in Figure 1, MCM signal is low in G2/M and increases sharply within ~20–40 min after release, reaching near-maximal levels by ~50–60 min. Insets show representative DNA-content profiles corresponding to each time point.

(B) Quantification of licensing and CDK regulatory markers during the same time course. The fraction of licensed origins at two origins (ARS1103 and ARS224) was measured by ChEC–qPCR (blue) and compared with the fraction of cells in G1 determined by flow cytometry (orange). Immunoblot analysis of Clb2 and Clb5 together with the CDK inhibitor Sic1 shows rapid loss of B-type cyclins and accumulation of Sic1 coincident with the onset of MCM loading, consistent with licensing occurring as CDK activity declines. qPCR results are available in Supplemental Table S5.

(C) Genome-wide kinetics of helicase loading. Heatmap of normalized MCM–ChEC–seq signal across 326 origins, aligned relative to the ACS as in (A) and ordered by the time to half-maximal MCM loading ($t_{½}$). As in Figure 1, most origins acquire MCM signal within the same narrow time window, demonstrating highly synchronous genome-wide helicase loading. MCM-ChEC signal and $t_{½}$ values for individual origins are provided in Supplemental Table S4.

(D) Reproducibility of origin-specific licensing kinetics. Comparison of the time to half-maximal MCM loading ($t_{½}$) for each origin between two independent experiments. Each point represents one origin (n = 326). The strong correlation (Pearson r = 0.88) indicates that origin-specific licensing kinetics are highly reproducible despite a modest offset in absolute timing between experiments.

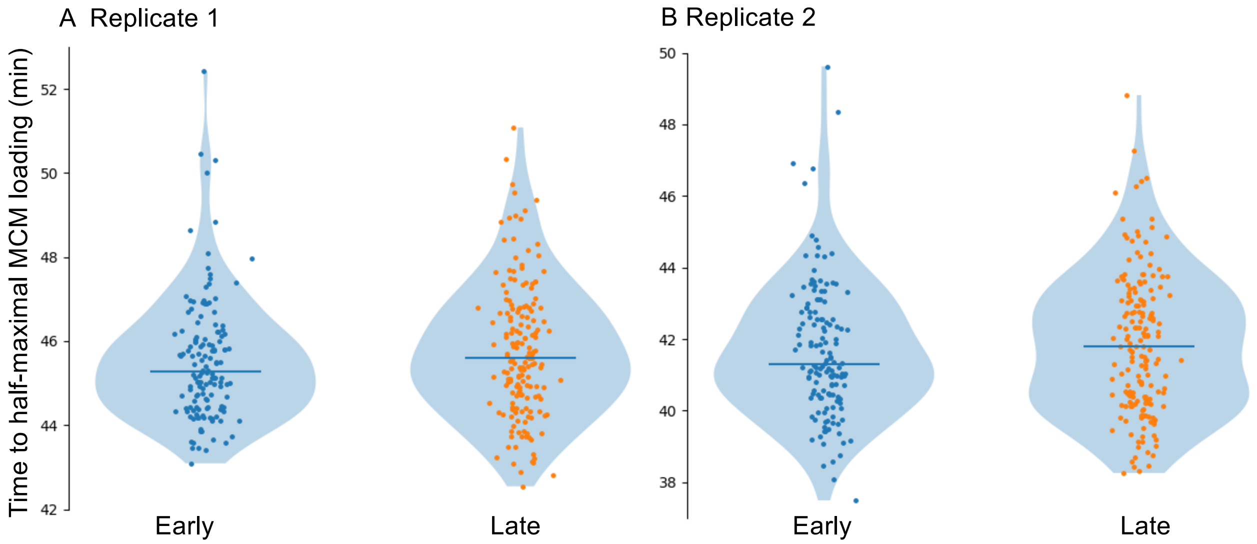

Figure S3. Early- and late-replicating origins exhibit similar licensing kinetics.

(A) Distribution of the time to half-maximal MCM loading (t½) for origins previously classified as early- or late-replicating in the experiment shown in Figure 1. Each point represents one origin, and violin plots show the distribution of t½ values within each class. Horizontal lines indicate the mean. Classification of early and late origins and t½ values for individual origins are provided in Supplemental Table S4.

(B) Same analysis as in (A) for the independent replicate experiment shown in Figure S2. Early- and late-replicating origins exhibit largely overlapping distributions of licensing kinetics in both experiments, indicating that replication timing classes do not differ substantially in the timing of MCM loading.

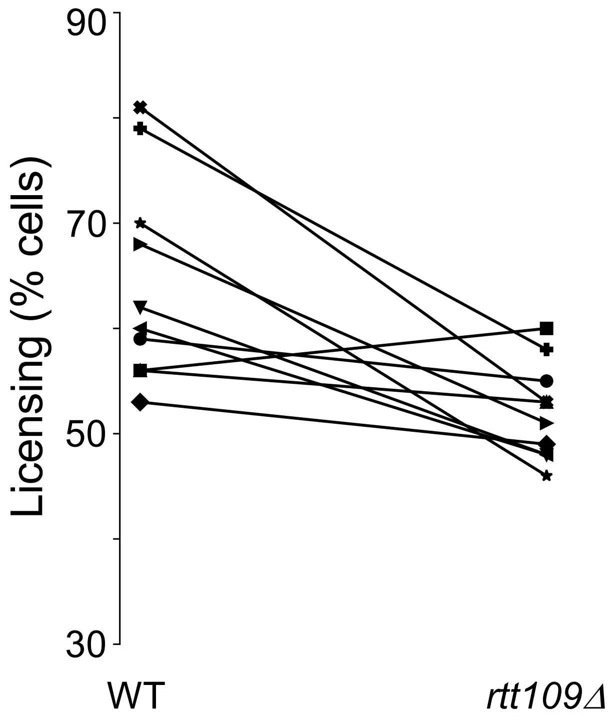

Figure S4. Independent replicate confirming reduced origin licensing in *rtt109*Δ cells.

MCM loading was measured by ChEC–qPCR at ten replication origins in wild-type and *rtt109*Δ cells in an independent experiment. As observed in Figure 2A, loss of H3K56 acetylation in *rtt109*Δ cells caused a modest but reproducible reduction in licensing relative to wild type (mean −19%; range −35% to +7%; paired two-tailed t-test p = 0.0046). Each line represents an individual origin. Individual qPCR values are provided in Supplemental Table S8.

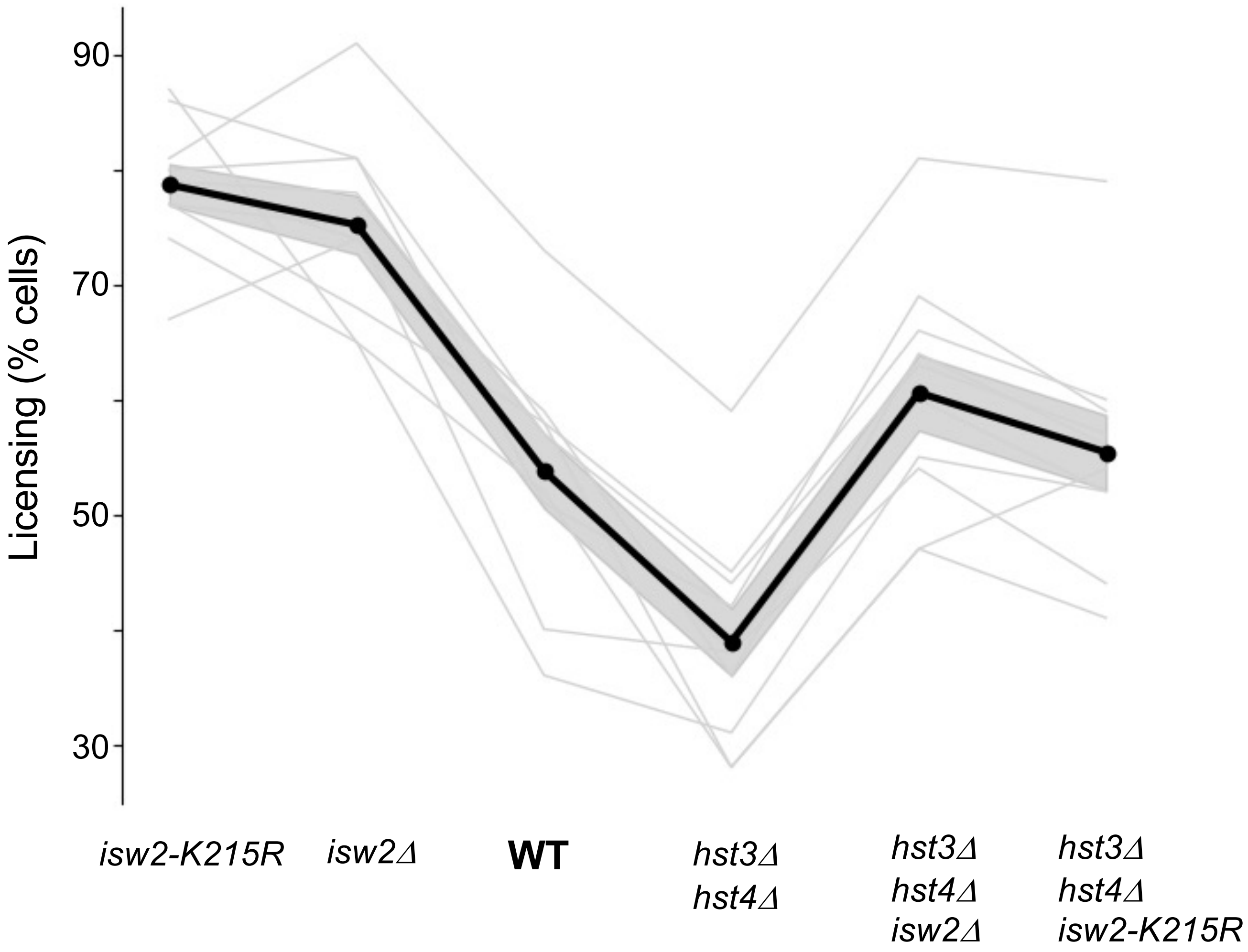

Figure S5. Independent biological replicate of the experiment shown in Figure 2D.

MCM loading was measured by ChEC–qPCR at ten replication origins in the indicated strains in an independent biological replicate. The same pattern observed in Figure 2 was reproduced: deletion of *ISW2* increased licensing relative to wild type, whereas *hst3*Δ *hst4*Δ cells exhibited a pronounced licensing defect that was strongly suppressed by deletion of *ISW2*. The ATPase-dead *isw2*-K215R allele phenocopied *isw2*Δ in both wild-type and *hst3*Δ *hst4*Δ backgrounds, indicating that catalytic remodeling activity of Isw2 is required for inhibition of origin licensing. Thin gray lines represent individual origins; the thick black line indicates the mean across origins, and the shaded region denotes ± SEM. Individual qPCR values are provided in Supplemental Table S6.

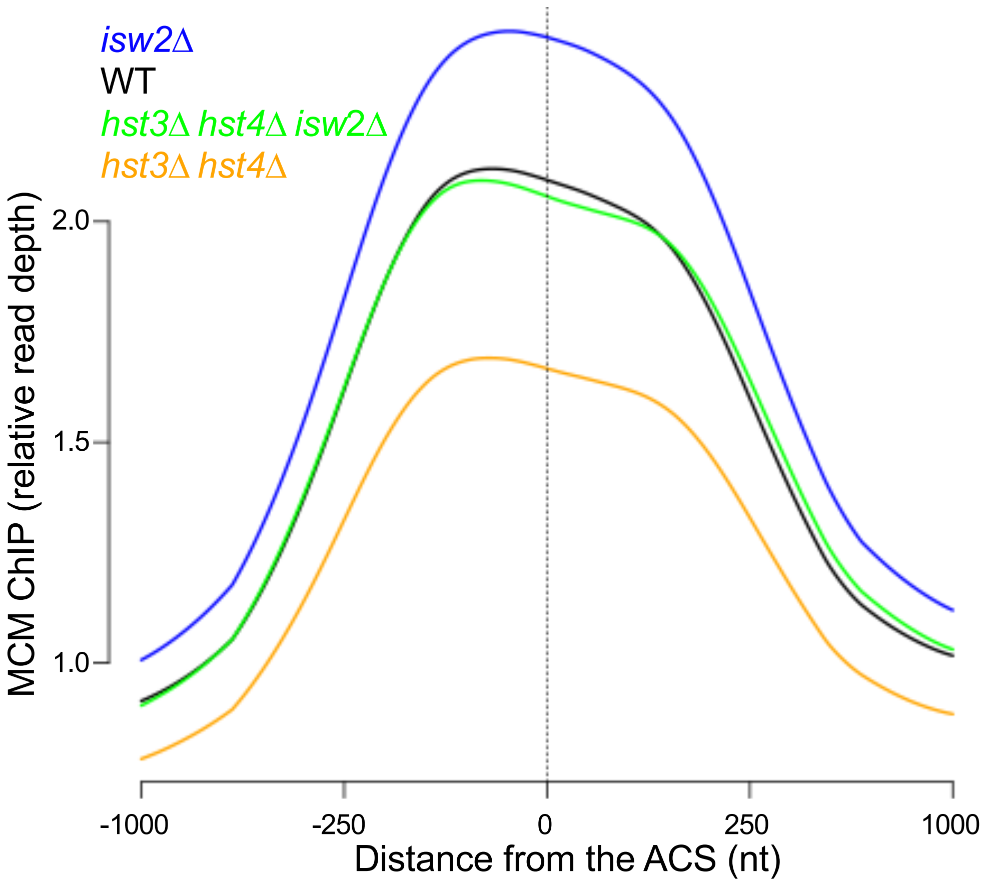

Figure S6. Genome-wide MCM ChIP confirms licensing differences across genotypes.

Average MCM ChIP signal across 326 replication origins aligned relative to the ARS consensus sequence (ACS) in the indicated genotypes (WT, *isw2*Δ, *hst3*Δ *hst4*Δ, and *isw2*Δ *hst3*Δ *hst4*Δ). Origin-associated MCM signal is reduced in *hst3*Δ *hst4*Δ, increased in *isw2*Δ, and restored in *isw2*Δ *hst3*Δ *hst4*Δ, consistent with the licensing differences measured by MCM–ChEC. An independent biological replicate yielded highly similar results; the corresponding values for both replicates are provided in Supplemental Table S11.

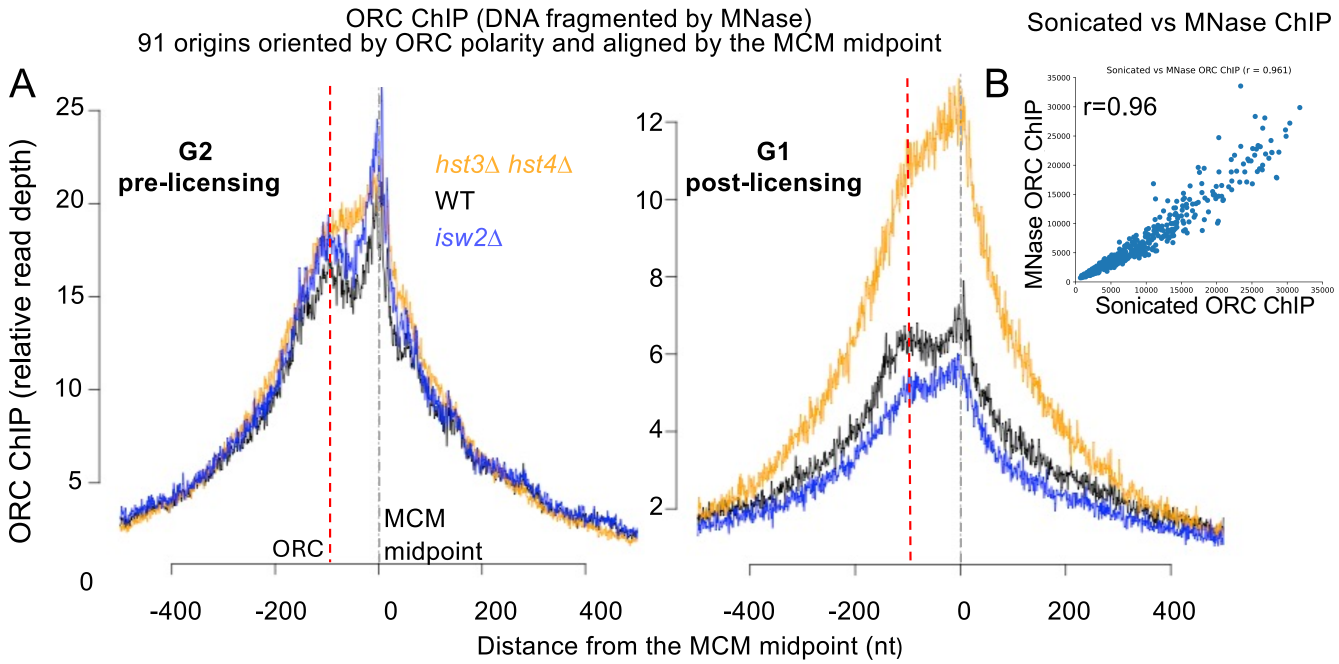

Figure S7. ORC ChIP profiles using MNase-fragmented chromatin

(A) Average ORC ChIP signal across 91 replication origins oriented by ORC polarity and aligned by the MCM loading midpoint in G2/M (pre-licensing) and G1 (post-licensing) cells of the indicated genotypes (WT, *isw2*Δ, and *hst3*Δ *hst4*Δ). Chromatin was fragmented using exogenous MNase digestion. As in the sonicated ChIP experiment shown in Figure 3, the ORC peak lies to the left of the MCM midpoint, consistent with the orientation of the curated origin set. Although the contours of the profiles differ from those obtained with sonicated chromatin, reflecting differences in fragmentation, the relative ORC occupancy patterns across genotypes are preserved. Relative ORC ChIP signal at each of the 91 origins for both fragmentation methods is provided in Supplemental Table S10.

(B) Correlation between ORC ChIP signals obtained from sonicated and MNase-fragmented chromatin across all origins and conditions (Pearson $r = 0.96$, $N = 546$), demonstrating strong agreement between the two fragmentation methods.

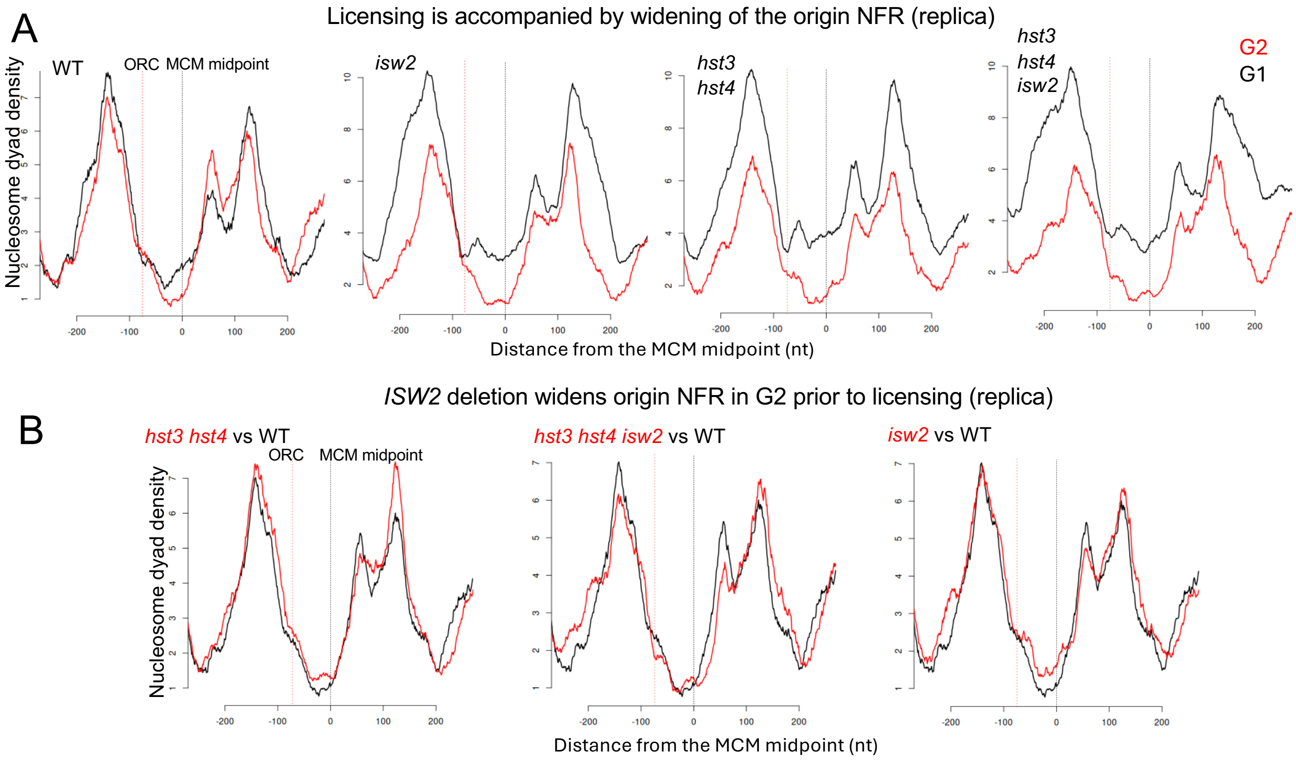

Figure S8. Independent biological replicate of nucleosome mapping at replication origins.

(A) Nucleosome dyad density profiles derived from MNase digestion in G2/M (red) and G1 (black) cells for the indicated genotypes across 91 oriented replication origins. Profiles are aligned to the MCM midpoint and oriented as in Figures 3–5. Vertical dashed lines indicate the ORC binding site and the MCM midpoint. As in Figure 5A, licensing in wild-type cells is accompanied by outward repositioning of nucleosomes flanking the origin NDR during G1.

(B) Direct comparison of nucleosome organization in G2/M prior to licensing. As in Figure 5B, deletion of *ISW2* widens the origin NDR and repositions flanking nucleosomes outward in the *hst3*Δ *hst4*Δ background. These results reproduce the nucleosome architecture changes observed in Figure 5.

Supplemental Tables

Supplemental Table S1. Yeast strains used in this study

Strain ID	Genotype	Source
16652	MATa his3 leu2 ura3 met15	This study
16964	MATa his3 leu2 ura3 met15 hmlαΔ::NAT MCM2-3xFLAG-MNase::KanMX	This study
16991	GP2 h− (L972) wild-type *Schizosaccharomyces pombe*	Gift from G. Smith
17000	GP2 h− (L972) MCM2-3xFLAG-MNase::KanMX	This study
17862	MATa his3 leu2 ura3 met15 hmlαΔ::NAT MCM2-3xFLAG-MNase::KanMX isw2Δ::Hyg	This study
17868	MATa his3 leu2 ura3 met15 hmlαΔ::NAT MCM2-3xFLAG-MNase::KanMX isw1Δ::Hyg	This study
17870	MATa his3 leu2 ura3 met15 hmlαΔ::NAT MCM2-3xFLAG-MNase::KanMX chd1Δ::Hyg	This study
17876	MATa his3 leu2 ura3 met15 hmlαΔ::NAT MCM2-3xFLAG-MNase::KanMX nhp10Δ::Hyg	This study
17878	MATa his3 leu2 ura3 met15 hmlαΔ::NAT MCM2-3xFLAG-MNase::KanMX rtt109Δ::Hyg	This study
17887	MATa his3 leu2 ura3 met15 hmlαΔ::NAT MCM2-3xFLAG-MNase::KanMX hst4Δ::Hyg hst3Δ::LEU2	This study
17913	MATa his3 leu2 ura3 met15 isw2Δ::Hyg	This study
17923	MATa his3 leu2 ura3 met15 hmlαΔ::NAT MCM2-3xFLAG-MNase::KanMX hst4Δ::Hyg hst3Δ::LEU2 isw2Δ::URA3	This study
17941	MATa his3 leu2 ura3 met15 hst4Δ::Hyg hst3Δ::LEU2 isw2Δ::URA3	This study
17945	MATa his3 leu2 ura3 met15 hst4Δ::KanMX hst3Δ::Hyg	This study
17947	MATa his3 leu2 ura3 met15 SIC1-TAP::HIS3MX6	Open Biosystems
17948	MATa his3 leu2 ura3 met15 CLB2-TAP::HIS3MX6	Open Biosystems
17949	MATa his3 leu2 ura3 met15 CLB5-TAP::HIS3MX6	Open Biosystems
17967	MATa his3 leu2 ura3 met15 ORC2-6xGly-3xFLAG::KanMX	This study
17976	MATa his3 leu2 ura3 met15 ORC2-6xGly-3xFLAG::KanMX isw2Δ::HIS3	This study
17980	MATa his3 leu2 ura3 met15 ORC2-6xGly-3xFLAG::KanMX hst3Δ::Hyg hst4Δ::NAT	This study
18100	MATa his3 leu2 ura3 met15 hmlαΔ::NAT MCM2-3xFLAG-MNase::KanMX hst4Δ::Hyg hst3Δ::LEU2 isw2-K215R	This study
18103	MATa his3 leu2 ura3 met15 hmlαΔ::NAT MCM2-3xFLAG-MNase::KanMX isw2-K215R	This study

Supplemental Table S2. Plasmids used in this study

Plasmid ID	Description	Source
pGZ108	pFA6a-3FLAG-MNase-kanMX6, Addgene #70231	Zentner et al., 2015
pGZ109	pFA6a-3FLAG-MNase-HIS3MX6, Addgene #70232	Zentner et al., 2015
pRS406-ISW2-GRT	K215R mutation in the ATPase domain of ISW2	Gelbart et al., 2005

Supplemental Table S3. Primers used for qPCR

Primer Name	Target	Sequence (5'→3')
Tor2_5P_qPCR	TOR2	GCACAAAGACCACAAAGTCG
Tor2_3P_qPCR	TOR2	GGGGATAGAGAACTAACAAAAGCA
ARS216_qPCR_Forward	ARS216	CCCTTGGTATACCTTTTCTGTG
ARS216_qPCR_Reverse	ARS216	CCCATCTTTAAGCTCTGCTGTG
ARS224_qPCR_Forward	ARS224	GCATAATGCCTTATTTTGTACC
ARS224_qPCR_Reverse	ARS224	GGCACATTCATACTTATCTTAGC
ARS315_qPCR_Forward	ARS315	GTGGGTATTTCCGTGTATATGG
ARS315_qPCR_Reverse	ARS315	GGTATTTGCAGTATTTTCTTGGC
ARS419_qPCR_Forward	ARS419	GAGCTTTAAACAAGGAGGAG
ARS419_qPCR_Reverse	ARS419	GATAACCGATGACTTGGCAAG
ARS605_qPCR_Forward	ARS605	CCCAGACACAGTCTTCATTTATAC
ARS605_qPCR_Reverse	ARS605	CATTCTGCAATAAAACCTGACC
ARS802_qPCR_Forward	ARS802	CACTTTACACCATACAATAACCAC
ARS802_qPCR_Reverse	ARS802	CTAATGTGAAGCCTGTGTAC
ARS1103_5P_qPCR	ARS1103	CACTTAACTTGTTATAATTCTCCC
ARS1103_3P_qPCR	ARS1103	CCATTCTGGTAGTTTTAATGTATTG
ARS1235_qPCR_Forward	ARS1235	CATGGAAGGGTTACTCACAACG
ARS1235_qPCR_Reverse	ARS1235	CGTATTAAACTAGATGACGAACAG
ARS1406_qPCR_Forward	ARS1406	GACGCATTATACAATCCTAC
ARS1406_qPCR_Reverse	ARS1406	GCCATTTTATATGTACATTCATC
ARS1427_qPCR_Forward	ARS1427	CAACCACAGTGGATATTTCCC
ARS1427_qPCR_Reverse	ARS1427	GCCATACGAATAAGGTGG
